# Impact of response shift on change in patient-reported outcomes: a systematic review

**DOI:** 10.1007/s11136-026-04307-8

**Published:** 2026-06-15

**Authors:** Mathilde G. E. Verdam, Jae-Yung Kwon, Lara Russell, Véronique Sébille, Mirjam A. G. Sprangers, Richard Sawatzky, The Response Shift –in Sync Working Group

**Affiliations:** 1 Department of Methodology and Statistics, Institute of Psychology, Leiden University, Leiden, The Netherlands; 2 Medical Psychology, Amsterdam UMC Location University of Amsterdam, Amsterdam, the Netherlands; 3 School of Nursing, Trinity Western University, 7600 Glover Road, Langley, BC V2Y 1Y1 Canada; 4 School of Nursing, University of Victoria, Victoria, BC Canada; 5 Institute On Aging and Lifelong Health, University of Victoria, Victoria, BC Canada; 6 MethodS in Patient-Centered Outcomes and HEalth ResEarch, CHU Nantes, INSERM, Nantes Université, Université de Tours, SPHERE, 44000 Nantes, France; 7 Amsterdam Public Health, Mental Health, Amsterdam, the Netherlands; 8 Centre for Advancing Health Outcomes, Providence Health Care Research Institute, Vancouver, Canada; 9 Institute of Health and Care Sciences, and Centre for Person-Centred Care (GPCC), Sahlgrenska Academy, University of Gothenburg, Gothenburg, Sweden

**Keywords:** Response shift, Patient-reported outcomes, Impact, Clinical relevance, Systematic review

## Abstract

**Purpose:**

The aim of this systematic review is to provide insight into whether response shift impacts the conclusions about change in patient-reported outcome measures (PROMs) in terms of statistical significance, magnitude, and decisions made.

**Methods:**

Response shift studies from Sawatzky et al. (2025; QLR) were analyzed: longitudinal quantitative studies that examined response shift using PROMs, published before May 2023. We determined whether: 1) impact of response shift was investigated, 2) information about change in the scores of a PROM (i.e., PROM-result) before and after taking response shift into account was provided, and 3) impact of response shift was evidenced in terms of statistical significance, magnitude, or decisions made.

**Results:**

A total of 173 response shift studies that included 943 PROM-results were evaluated. 55% of studies (N = 96) investigated impact of response shift and information about impact was available for 51% of studies (N = 89). The corresponding percentages based on PROM-results were 47% (N = 446) and 53% (N = 502), respectively. Impact of response shift was evidenced in 69% of studies (N = 61) and 41% of PROM-results (N = 207), where impact on statistical significance and/or magnitude of change were most often evidenced (49–53% of studies and 20–31% of PROM-results), whereas impact on decisions was evidenced less often (8% and 2% respectively).

**Conclusion:**

About half of the studies addressed impact of response shift and showed evidence of impact, however, impact on decisions was rarely addressed. Future research should focus on evaluating impact of response shift on conclusions about change in PROMs, especially in the context of healthcare decision-making.

## Introduction

**Fig. 1:**
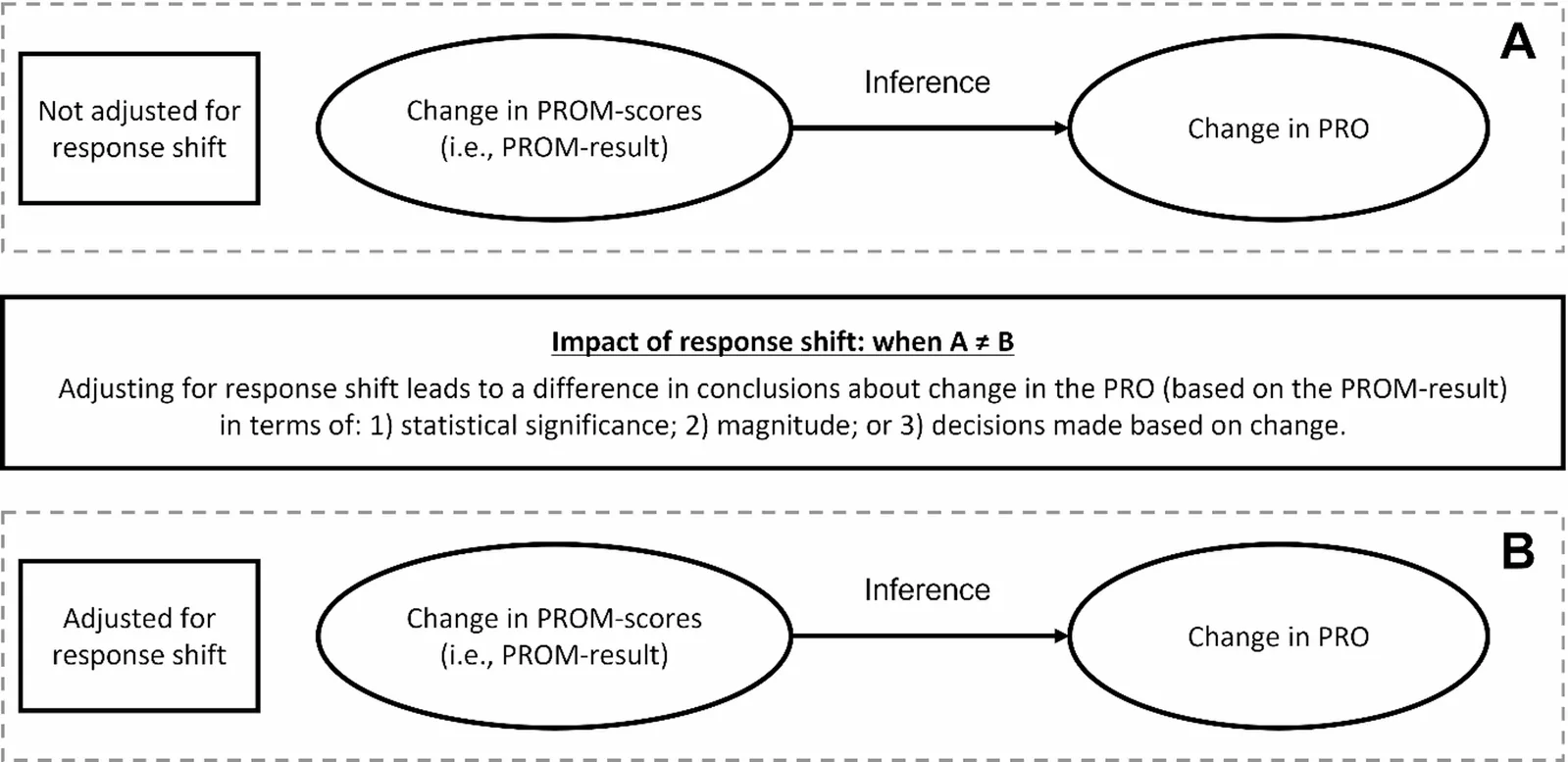
Impact of response shift. *Notes*: Panel A shows change in the PRO when response shift is not considered, whereas panel B shows change in the PRO when adjusted for response shift. Impact of response shift is defined as a difference in conclusions about change between (**A**) and (**B**)

Patient-reported outcome measures (PROMs) are increasingly used to acquire information about health and quality of life from the patient’s perspective. PROMs can provide insight into patients’ perceived health trajectories and are important endpoints in studies to evaluate health care. A challenge with the interpretation of change in PROM-scores (i.e., PROM-result) is that the meaning of patients’ self-evaluation may change. That is, observed change on a PROM may not be fully attributable to change in the target construct that the PROM aims to measure (i.e., the patient-reported outcome (PRO), e.g., health or quality of life), because of a change in the meaning of the subjective evaluation. This effect is also known as response shift [1–3]. Occurrences of response shift, when ignored, may result in biased conclusions about changes in PROs. For example, response shift may affect PROM-results in a way that could lead to either under- or over-estimation of treatment effectiveness. Reviews of response-shift studies estimated the prevalence of response shift to be between 71–87% [4, 5]. However, there is a gap in our knowledge about the extent to which occurrences of response shift also impact the interpretation of PROM-results, and therefore the inferences made on change in the PROs. Here, impact of response shift is defined as the difference in the conclusion(s) about change in the PRO (based on the PROM-result) before and after taking response shift into account (see Fig. 1).

Knowledge about *impact* is important because evidence of response shift is not necessarily the same as evidence of impact. For example, a response shift effect may not be statistically significant, but change in PROM-scores may become statistically significant when adjusted for this insignificant response shift effect, thereby impacting the inference about change in the PRO. To exemplify, Addington-Hall et al. [6] found no statistically significant difference between a baseline and retrospective (then-test) score on a quality-of-life PROM (i.e., no response shift), but also found that change was significant using the retrospective measure (i.e., adjusting for response shift) and not with the conventional baseline measure. Another possibility is that response shift may impact the direction of change, thereby altering the conclusions about the PROM-result (e.g., an increase instead of a decrease in symptoms). To illustrate, Gillison et al. [7] investigated change in quality of life in adolescents and found that taking a small response shift effect into account resulted in an improvement instead of a deterioration in the domain ‘mood and emotions’. Alternatively, statistically significant response shift effects may not necessarily impact conclusions about change in PROMs when adjusting for response shift. For example, Müller et al. [8] found response shift in patients’ fatigue scores after cognitive behavioural therapy, but these effects did not impact the conclusions about treatment effectiveness (i.e., treatment effects remained statistically significant and of the same magnitude).

Impact of response shift thus provides insight into the clinical relevance of response shift over and beyond the mere evidence of the occurrence of response shift itself. However, the extent to which response shift impacts conclusions about PROM-results is unknown. Preliminary evidence indicates that response shift impacted the assessment of treatment effectiveness in a review of ten randomized clinical trials (RCTs) [9]. It was concluded that response shift obfuscated treatment effectiveness in seven out of the ten included RCTs. However, impact was evaluated based on whether “the treatment(s) being assessed in the clinical trial have a more or less beneficial impact on outcomes when response-shift effects were considered” (p. 3), but no details were given as to what “more or less beneficial” means. Therefore, a more detailed and extensive assessment of impact of response shift is needed.

The Response Shift – in Sync Working Group [10] was established to provide a critical and comprehensive appraisal of the response-shift work to date and included a systematic review [5] and meta-regression analysis [11] on response shift effects in quantitative health research using PROMs. In this earlier work we have addressed the prevalence and magnitude of response shift effects (150 studies [5]) and the study-characteristics that explain variability in response shift detection and magnitude (173 studies [11]). The current study brings this work a step further by reviewing the same 173 studies and investigating whether response shift affected the conclusions made based on PROM-results in terms of (1) statistical significance, (2) magnitude, and (3) decisions made (e.g., determining whether a treatment is effective or not). In so doing, we provide a systematic approach to investigating impact of response shift that can be applied to all response shift studies. Our overarching aim is to offer insight into the extent to which response shift effects impact PROM-results, and hence the importance or (clinical) relevance of response shift for conclusions about change in PROs.

## Methods

### Selection of studies

**Fig. 2:**
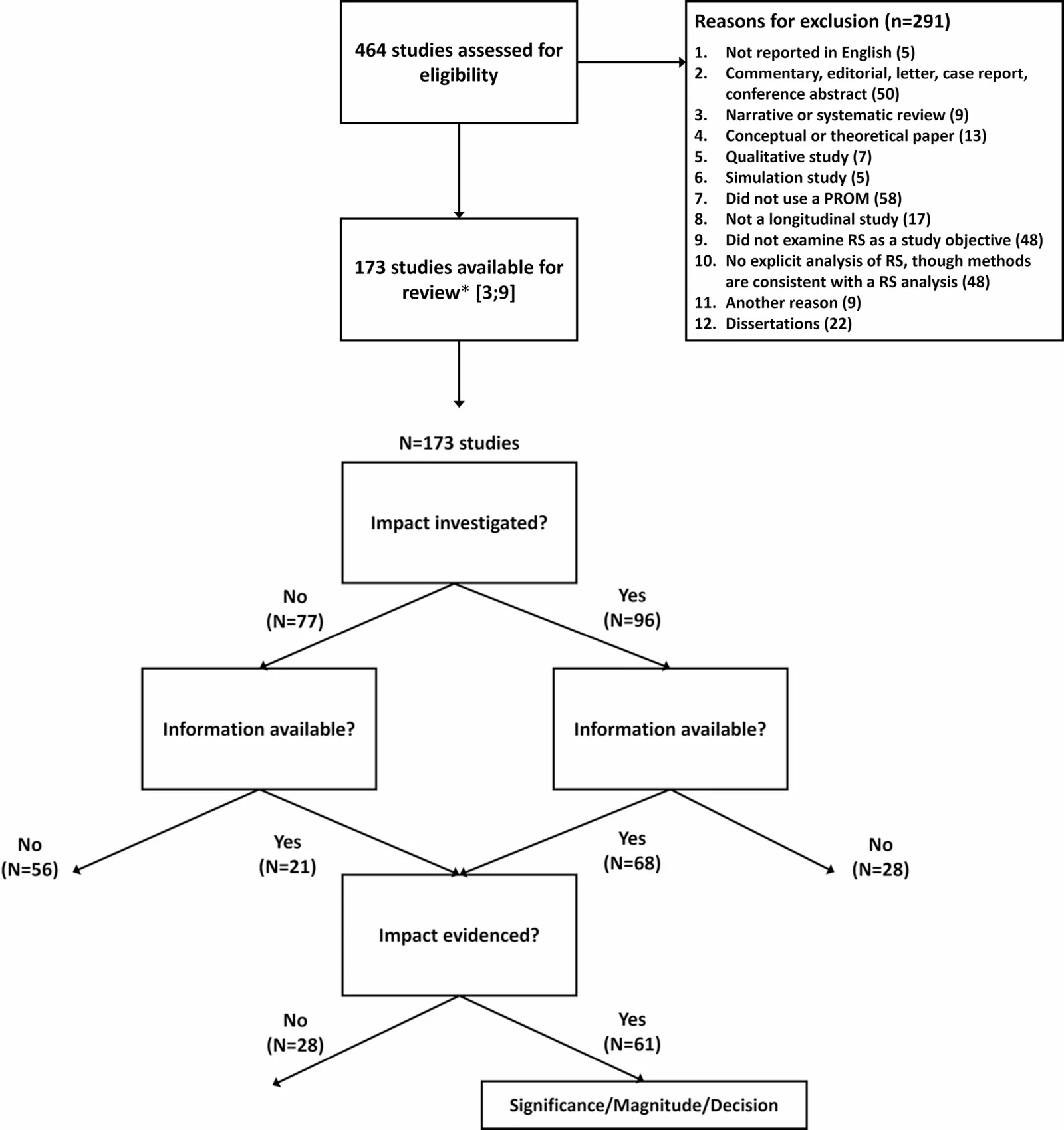
Data screening and extraction diagram for the investigation of impact, information about impact and evidence of impact of response shift. *Notes*: Studies on response shift were identified by searching the following library databases; **a** MEDLINE, PSYCINFO, and CINAHL using the EBSCO interface; **b** EMBASE using the OVID interface; **c** Social Science Citation Index using the Web of Science interface; and **d** Dissertations & Theses Global using the ProQuest interface. All searches were conducted using the same combination of the following terms and corresponding abbreviations in all indexed fields: “response shift” OR “longitudinal measurement invariance” OR “retrospective bias” OR “longitudinal differential item” OR “longitudinal DIF.” RS = response shift. PROM = patient-reported outcome measure, resulting in the identification of 4038 publications. *Of the 173 studies included in the review stage of the meta-regression, there were 2 studies excluded at the stage of analysis due to dependency with data from other studies. See the original manuscripts for more details about the search strategy and article selection [5;11]

We used the same selection of 173 papers as in the meta-regression analysis on response shift effects [11]. Eligibility criteria were all longitudinal quantitative studies that examined response shift using a PROM. See Fig. 2 for more details about the search strategy, exclusion criteria, and data extraction.

### Data extraction

Table 1 provides an overview of the extracted data available from previous work [5;11] relevant for the current study. For data extraction regarding information about impact of response shift, the included studies were assigned in equal proportions to one of two team members (MV, JK). Ambiguities were discussed among team members to achieve agreement. All data extraction was subsequently reviewed by one team member (MV) to ensure consistency. Study-level results were extracted using the EPPI reviewer application [12]. Results on impact of response shift are evaluated at the study-level, overall and separate for each response shift method, but irrespective of there being multiple samples, PROMs and target constructs (e.g., multiple target constructs measured by the same multidimensional PROM) within the same study. For studies that reported on multiple PROM-results, we additionally e information about impact of response shift on PROM-results separately for each independent sample, PROM and target construct.xtracted We aggregated results at the level of the PROM-result and therefore did not distinguish impact from different types of response shift (i.e., recalibration, reprioritization and reconceptualization) or for multiple measurement occasions (e.g., when PROM-scores were evaluated at multiple occasions, impact on change at any one (or more) of the occasions was taken to be indicative of impact on the PROM-result). Information about impact was only extracted for independent samples within each study (i.e., subsamples were ignored due to inherent dependencies with the overall sample). Extracted data are available at https://osf.io/q9erk/.

**Table 1:** Extracted data available from previous work [5;11] that was used to describe the results on impact of response shift

Variable	Description
Study	Unique response-shift studies as identified in the original systematic review [3;9] were used to stratify results on impact of response shift across studies
Response shift methods	We distinguish between design-based methods, latent variable methods, regression methods, and study-specific methods. This classification of methods is based on previous work [13] and detailed descriptions of response shift operationalization for each method are provided elsewhere [5; 13]
Design-based methods	*Then-test* (i.e., an additional measurement at follow-up occasion with the instruction to re-evaluate baseline functioning) *Individualized methods* (e.g., SEIQOL, patient-generated index, Cantrill’s ladder) *Other design-based methods* (e.g., appraisal, change in importance ratings, vignette studies)
Latent variable methods	*Structural Equation Modeling (SEM)* (e.g., Oort’s method, Schmitt’s method) *Item Response Theory (IRT) or Rasch models* (i.e., latent factor models based on IRT or Rasch measurement theory)
Regression methods	*Regression methods with classification* (e.g., regression-tree methods, random forest regression, growth mixture models) *Regression methods without classification* (e.g., relative important analysis)
Other study-specific methods	Methods that are unique to a particular study and have not been applied in other studies. This includes various combinations of other design-based methods and other statistical methods
Sample	Within studies, there may be a single unique sample, there may be multiple unique samples, and there may be (multiple) subsamples from an overall sample investigated within the same study. We include unique samples within studies (i.e., one or multiple unique samples) and exclude subsamples from an overall sample within the same study. Dependencies between samples across studies (e.g., when the same sample is investigated in more than one study) are ignored
PROM-characteristics	We distinguish between different PROMs and PROM domains (i.e., target constructs) by distinguishing the name(s) of PROM(s) and name(s) of the PROM domain(s) used for response shift investigation

Results on impact are reported at the study-level (overall, and separate for each response shift method), and at the level of PROM-results, i.e., stratifying not only over studies, but also for unique samples, response shift methods, PROMs, and PROM domains (i.e., target constructs)

**Investigation of impact: **First, we determined whether the study investigated impact of response shift (yes/no). When a study reported on any type of impact of response shift on the interpretation of PROM-results, we labeled this as “yes” for *impact investigation*. This first criterion includes investigations of impact that are consistent with our definition of impact (i.e., a difference in conclusion(s) made about change in the PRO (based on PROM-results) before and after taking response shift into account), as well as alternative approaches for investigating impact that are not consistent with our definition (e.g., the relationship between response shift and other variables). This broader criterion was chosen to acknowledge the different approaches for investigating impact of response shift.

**Information about impact:** Second, we determined whether the study provided *information* about change in the estimated values of the PROM-scores and the corresponding target construct(s) before and after taking response shift into account (yes/no). This information criterion allows for a more precise evaluation of evidence on impact as information on evidence was only available for studies that provided enough (statistical) information about impact of response shift (see Fig. 2 for a visual representation of the data extraction). That is, some studies do investigate impact of response shift but do not provide information about change in PROM-scores before and after taking response shift into account (i.e., information about impact on statistical significance, magnitude or decisions was not available). Conversely, some studies do not explicitly investigate impact of response shift but do provide information about (un)adjusted change in PROM-scores.

**Evidence on impact:** Third, *impact of response shift* was indicated when a difference was found in conclusions about change in the values of the target construct (as measured by the PROM) before and after taking response shift into account in one or more of the following ways:

1. Significance: a difference in the statistical significance of change (as determined following criteria provided by the authors of the original studies, if available, and otherwise with alpha = .05) in the target construct (i.e., whether change in target construct becomes significant or is no longer significant when adjusted for response shift);
2. Magnitude: a difference in the magnitude of change in the target construct (i.e., in terms of interpretation of effect-size estimates as small/medium/large according to conventional rules of thumb [14] or whether change is below/above a threshold for the minimally important difference, as defined by the author);
3. Decision: a difference in decisions made based on change in the target construct (e.g., in terms of treatment- or cost-effectiveness).

Table 2 provides a detailed overview of the operationalizations of impact of response shift that are consistent with our definition of impact, for different response shift methods. If impact of response shift was not explicitly addressed by the authors, information about evidence of impact was derived based on the provided information, if available (i.e., when statistical significance and/or magnitude of change in the target construct before and after taking response shift into account was provided or could be calculated).

**Table 2:** Operationalizations of impact of response shift for different response shift methods

	Operationalizations of impact of response shift
Then-test method:	Using the then-test methodology, change in the target construct before taking response shift into account is operationalized as the difference between pre- and post-test scores (which is equal to change in observed (PROM) scores, i.e., no response shift assumed, all observed change is assumed to be change in the target construct); whereas the difference between then- and post-test scores is change in the target construct after taking response shift into account. The difference between pre-post change scores and then-post change scores is taken to be indicative of whether, and the extent to which, response shift impacts change in the target construct
Latent variable methods:	For latent variable methods (i.e., structural equation modeling (SEM), item response theory (IRT), and Rasch measurement theory (RMT)), change in the target construct is the change in the underlying latent variable(s). Thus, the differences in estimated change in the underlying latent variable(s) before and after taking response shift into account is indicative of whether, and the extent to which, response shift impacts change in the target construct(s) [15]
All other methods:	For other methods (i.e., appraisal methods, individualized methods, vignettes studies, relative importance analyses, classification and regression-tree methods, and random forest models) adjusting for response shift in the target construct is not straightforward (see [13] for more information). It is therefore not possible to determine a-priori how impact of response shift is operationalized with these methods (if at all). Nonetheless, we reviewed papers that applied these other response shift methods and extracted the available information, if applicable

When means and standard deviations of the estimated values of PROM-results and the corresponding target construct(s) (or the scores on associated separate measurements) were available, standardized mean differences (Cohen’s *d*) for change scores could be calculated [14]. For some studies we first had to transform medians, interquartile ranges (IQR), or confidence intervals (CIs) into corresponding means and standard deviations [16; 17]. Statistical significance of change-scores was determined using 95% CIs. All calculations that were used for evaluated studies are available through https://osf.io/q9erk/

## Results

### Impact of response shift: study-level results

Of the 173 response shift studies, 96 studies (55%) investigated impact of response shift (see Fig. 2 and Table 3). Of these 96 studies, 64 (67%) studies investigated impact by comparing inferences and/or conclusions about change in the value of the target construct(s) (as measured by the PROM) before and after taking response shift into account, whereas 32 studies (33%) investigated impact using alternative approaches that are not consistent with our definition (Table 4 provides a description of these impact investigations).

**Table 3:** Number of studies that investigated impact, and/or provided information from which impact could be inferred; overall and per response shift method

Impact investigated?		Yes (N = 96; 55%)		No (N = 77; 45%)	
Information about impact available?		Yes	No	Yes	No
Total #nr of studies		N (%)^1^	N (%)^1^	N (%)^1^	N (%)^1^
Design-based methods:	98^*^				
Then-test	84	50 (60%)	3 (4%)	21 (25%)	10 (12%)
Individualized methods	12	2 (17%)	4 (33%)	–	6 (50%)
Other methods	12	–	1 (8%)	–	11 (92%)
Latent variable methods:	58^*^				
SEM	56	17 (30%)	14 (25%)	–	25 (45%)
IRT/RMT	4	2 (50%)	1 (25%)	–	1 (25%)
Regression methods:	27^*^				
With classification	11	–	2 (18%)	–	9 (82%)
Without classification	17		4 (24%)	–	13 (76%)
Other study-specific methods	5	–	–	–	5 (100%)
Overall	173^*^	68 (39%)	28 (16%)	21 (12%)	56 (32%)

SEM, structural equation modeling. IRT/RMT = item response theory / Rasch measurement theory. ^*^ Because some studies employ multiple methods the total(s) are not a sum of the number of studies in the individual associated categories. The final row (Overall) shows the total number of studies but is not an addition of the associated columns as one study can employ multiple methods. ^1^ The denominator for the percentage is the total number of studies in the same row. The row percentages do not always add up to 100% due to rounding

**Table 4:** Description of alternative^1^ approaches for investigating impact of response shift, per response shift method

	N studies	Description of impact investigations
Design-based methods:	14	
**Then-test**	9	Of the 9** then-test** studies, four studies [18–21] only compared (un)adjusted change scores relative to each other (but not in terms of changes in conclusions based on these scores). One study [22] focused on post-test scores (but not change scores), another study [23] used regression-based analyses to relate change in PROM-scores and response shift values, and three studies investigated impact of response shift on other variables: time-to-deterioration [24], determination of minimal important difference [25], and quality-of-life adjusted life-years [26]. Of the four studies that used **individualized methods**, two studies used subgroup-analyses to compare PROM-results for participants with and without response shift [27, 28] and two studies investigated the relationship between response shift and PROM-results but did not (provide sufficient information to) compare PROM-results before and after taking response shift into account [29, 30]. The latter also applied to one study that used** another method** [31]
**Individualized methods**	4	
**Other methods**	1	
Latent variable methods:	13	
**SEM**	12	Of the twelve studies that used** SEM**, there were eleven studies that investigated impact at the level of the observed indicators, but not at the level of the latent (target) construct (cf. [32]), and one study [33] used an additional simulation study to investigate impact of ignoring response shift on the results of paired t-tests. One** IRT/RASCH** study [34] implemented an additional IRT analysis to investigate the impact of response shift on change in the latent target construct
**IRT/RASCH**	1	
Regression methods:	5^*^	
**With classification**	2	Of the two studies that used regression methods** with classification**, one study used subgroup-analyses based on response shift results to describe differences in PROM-results [35], and other study used a regression-based methods to relate an operationalization of response shift to (change in) values of the target construct or PROM [36]. The latter also applied to the four studies that used regression methods **without classification** [36–39]
**Without classification**	4	
Other study-specific methods	0	–
Overall	32^*^	

^1^Alternative to comparing inferences and/or conclusions about change in the value of the target construct before and after taking response shift into account. SEM; structural equation modeling, IRT/RMT; item response theory/Rasch measurement theory. ^*^Because some studies employ multiple methods the total(s) are not a sum of the number of studies in the individual associated categories

Information about impact of response shift (i.e., impact on statistical significance, magnitude and/or decision) was available for 68 (71%) of the 96 studies that investigated impact. In addition, for 21 studies (12% of N = 173) information about impact of response shift was extracted from the provided statistics, even though impact was not explicitly investigated. Information about impact was not available for 84 (49%) of the 173 studies; 28 studies (16%) investigated impact but did not provide information about impact, and 56 studies (32%) did not investigate impact and did not provide information from which impact could be inferred. Thus, information about impact was available for (and therefore evidence of impact could be evaluated in) 89 studies (51%). The population-, study design-, and PROM characteristics of these 89 studies are provided in Table 5.

**Table 5:** Descriptive information on population-, study design- and PROM-characteristics (for N = 89 studies; and N = 502 PROM-results) for which impact of response shift could be evaluated

	N Studies	N PROM-results
Population characteristics		
Sex		
Mixed	73	356
Only female	7	85
Only male	6	47
Other/Unknown	3	14
Age		
Mostly adults	61	361
Mostly older adults	7	38
Mostly children/adolescents	17	81
Other/Unknown	4	22
Medical condition		
Yes: Cancer	28	228
Yes: Orthopedic	6	24
Yes: Stroke	2	4
Yes: Mental Health	7	20
Yes: Other	42	197
No	6	29
Intervention		
No/Unclear	20	130
Yes: Medical	49	277
Yes: Psychological	17	59
Yes: Other/Unspecified	3	36
Study design characteristics		
Design		
Observational	71	434
Experimental	18	68
Sample Size		
Q1 (< 57)	31	131
Q2 (57—254)	49	210
Q3 (255 – 410)	16	113
Q4 (> 411)	10	48
Primary data analysis		
No	68	400
Yes	21	102
PROM characteristics		
PROM type		
Generic PROMs	39	192
Disease-specific PROMs	37	215
Individualized/other PROMs	32	95
PROM domain		
General health/QOL	54	80
Physical	57	203
Psychological	51	117
Social	25	42
Pain	24	30
Other	17	30

The descriptive information and associated categories are consistent with those included in the original systematic review [3]. The number of studies within a category does not always add up to 89 as there may be multiple samples/methods/PROMs within studies with different characteristics

The results with regards to evidence of response shift are presented in Table 6 and show that 61 studies (69% of N = 89) evidenced impact in at least one of the target constructs under investigation in at least one of the three ways (significance, magnitude, or decision). A difference in statistical significance and a difference in magnitude of change was evidenced in a similar number of studies (44 and 47 respectively; or 49% and 53% of N = 89). However, there were only 7 studies (8% of N = 89) that evidenced a difference in decisions. Illustrative examples on the different types of impact evidenced are provided in Table 7. Comparison between *detection* of response shift and *impact* of response shift (see Table 8) shows that of the 75 studies that detected response shift, there were 20 studies (27%) that did not evidence impact of response shift. Of the 14 studies that did not detect response shift effects, there were 6 studies (43%) that did show evidence of impact of response shift.

**Table 6:** Total number of studies that investigated impact, provided information about impact, and evidenced impact of response shift; overall and per response shift method

	Total #nr of studies	Impact investigated^3^	Information available^3^	Impact Evidenced			
				Overall^4^ N (%)^2^	Significance N (%)^2^	Magnitude N (%)^2^	Decision N (%)^2^
	N	N (%)^1^	N (%)^1^				
Design-based methods:	98^*^						
Then–test	84	53 (63%)	71 (85%)	56 (79%)	42 (59%)	43 (61%)	6 (8%)
Individualized methods	12	6 (50%)	2 (17%)	1 (50%)	1 (50%)	1 (50%)	0 (0%)
Other methods	12	1 (8%)	0 (0%)	–	–	–	–
Latent variable methods:	58^*^						
SEM	56	31 (55%)	17 (30%)	5 (29%)	1 (6%)	3 (18%)	1 (6%)
IRT/RMT	4	3 (75%)	2 (50%)	0 (0%)	0 (0%)	0 (0%)	0 (0%)
Regression methods:	27^*^						
With classification	11	2 (18%)	0 (0%)	–	–	–	
Without classification	17	4 (24%)	0 (0%)	–	–	–	–
Other study–specific methods	5	0 (0%)	0 (0%)	–	–	–	–
Overall	173^*^	96 (55%)	89 (51%)	61 (69%)	44 (49%)	47 (53%)	7 (8%)

SEM; structural equation modeling, IRT/RMT; Item response theory/Rasch measurement theory. ^*^ Because some studies employ multiple methods, the total(s) are not a sum of the number of studies in the individual associated categories. ^1^The denominator for the percentage is the total number of studies in the same row. ^2^The denominator for the percentage is the number of studies that provided information about impact in the same row. ^3^Note that some studies do investigate impact but do not provide information about impact, and vice versa (see Table 3). ^4^Impact evidenced in terms of a difference in statistical significance, magnitude and/or decisions before and after taking response shift into account

**Table 7:** Illustrative examples of response shift studies and the types of impact of response shift evidenced

Study description	Impact of response shift evidenced
Addington-Hall et al. [6]	
**Study design:** Thirty-five patients receiving hospice care filled in a QOL outcome measure (the St Christopher’s Index of Patient Priorities (SKIPP)) at two time-points (T1 and T2) **Response shift investigation:** Response shift was investigated by comparing T1 scores with retrospective scores assessed at T2 (then-test methodology) **Result:** They found no statistically significant response shift	**Significance: Yes.** The comparison between T1 and T2 QOL-scores (without adjusting for response shift) was not statistically significant (with alpha = .05), but the comparison between retrospective and T2 QOL-scores (i.e., adjusted for response shift) was significant **Magnitude: No.** Results on magnitude were derived from the provided statistics (i.e., median and IQR) and showed that both unadjusted and adjusted change-scores were of small size **Decision: No.** There was no mention of possible impact of response shift on decisions made based on the reported changes in QOL
Gillison et al. [7]	
**Study design:** A total of 356 adolescents completed a QOL measure (the Kidscreen) on two occasions **Response shift investigation:** Response shift was investigated with then-test and SEM methodology in ten different domains of QOL. Results on impact were only available for then-test methodology **Result**: They found statistically significant response shift in three QOL domains: ‘peers’, ‘mood and emotions’, and ‘autonomy’	**Significance: Yes.** For four domains of QOL the statistical significance (with alpha = .05) of the change in the domain-scores was different after adjusting for response shift. Changes in the domains ‘school’, ‘peers’ and ‘autonomy’ were no longer significant when adjusting for response shift, whereas change in the domain ‘bullying’ was only significant when adjusting for response shift **Magnitude: Yes.** The domains for which impact on statistical significance was indicated also showed impact on magnitude, where the size of the change-score was small and became negligible or was negligible and became small **Decision: Yes.** For the domain ‘mood and emotions’ a statistically significant improvement was found when adjusting for response shift, whereas without adjustment for response shift a significant finding of deterioration was found
Müller et al. [8]	
**Study design:** Data of three randomized controlled trials testing the efficacy of cognitive behavioural therapy (CBT) in individuals with chronic fatigue syndrome (CFS, n = 222), cancer (n = 123), and diabetes (n = 107) were re-analysed. Fatigue severity was measured with Checklist Individual Strength (CIS) at two occasions **Response shift investigation:** Response shift was investigated with SEM methodology in both control and intervention groups **Result:** They found one statistically significant (reprioritization) response shift in each trial, only in the CBT-groups	**Significance: No.** There was no evidence that changes in the estimated scores of fatigue severity changed in terms of statistical significance (with alpha = .05) after taking response shift into account **Magnitude: No.** Changes in the estimated scores of fatigue severity were of similar size both before and after taking response shift into account **Decision: No.** The authors conclude that response shift did not have impact on the intervention effect
Zhang et al. [21]	
**Study design:** Health-related quality of life was assessed with the EuroQol five-dimensional questionnaire (EQ-5D) and the six-dimensional health state short form (SF-6D) in 74 patients undergoing total knee replacement at three occasions, at baseline, 6 and 18 months after surgery **Response shift investigation:** Then-test assessments were administered at 18 months, to investigate response shift as compared with measures from baseline and compared to measures at 6 months **Result:** Response shift was indicated at baseline (both EQ-5D and SF-6D) and at 6 months (only SF-6D)	**Significance: No.** There was no evidence that the statistical significance (based on own calculations with alpha = .05) of change scores in SF-6D and EQ-5D were different before and after response shift into account **Magnitude: Yes.** After adjustment for response shift, the change in SF-6D scores between 6 and 18 months was no longer considered as clinically minimally important (according to the authors). Also, results on magnitude derived from the provided statistics (i.e., median and IQR) showed that change in EQ-5D scores between baseline and 18 months became larger after taking response shift into account **Decision: Yes.** The authors conclude that adjustment for response shift would lead to a substantial increase in the cost-effectiveness of treatment. Using example calculations, they show that the impact of response shift in SF-6D scores on the cost-utility ratio is as high as US$29,167 per quality-adjusted life-year (QALY), and $26,511 per QALY for the EQ-5D. They further describe that this could potentially impact decisions on the approval and subsidy of interventions and show that for the SF-6D the adjustment would yield a cost-utility ratio that is below a threshold of cost-effectiveness (whereas the unadjusted cost-utility ratio was not)
Bulteau et al. [50]	
**Study design:** A secondary analysis of data from an RCT on patient (n = 170) with Major Depressive Disorder (MDD) treated by pharmacotherapy (venlafaxine), repetitive Transcranial Magnetic Stimulation (rTMS), or both. Depression was measured with the 13-item short form Beck Depression Inventory (BDI-13) at two occasions **Response shift investigation:** Response shift was investigated with SEM methodology in the three intervention groups separately **Results:** In one group (venlafaxine + placebo rTMS) there was indication of response shift	**Significance: No.** The estimated change in depression scores was statistically significant (with alpha = .05), before and after adjusting for response shift, in all three groups **Magnitude: No.** The estimated change in depression across time was of similar size, before and after taking response shift into account, in all three groups **Decision: Yes.** One of the comparisons of treatment effects between groups became statistically significant (with alpha = .05) after adjusting for response shift. Specifically, the treatment effect in the venlafaxine + placebo rTMS group as compared to the rTMS + venlafaxine placebo group, was only found to significantly larger after taking response shift into account

Please refer to the original studies for many other noteworthy details about the population, study design, and PROM-characteristics

**Table 8:** Response shift detection versus impact of response shift

Response shift detected?		Yes N studies = 75 (84%)^1^ N PROM-results = 265 (53%)^1^		No N studies = 14 (16%)^1^ N PROM-results = 237 (47%)^1^	
Impact of response shift evidenced?		Yes	No	Yes	No
	Total #nr	N (%)^2^	N (%)^2^	N (%)^3^	N (%)^3^
#nr of studies	89	55 (73%)	20 (27%)	6 (43%)	8 (57%)
#nr of PROM-results	502	147 (55%)	118 (45%)	60 (25%)	177 (75%)

^1^The denominator for the percentage is the total number of studies or PROM-results. ^2^The denominator for the percentage is the total number of studies or PROM-results for which response shift was detected. ^3^The denominator for the percentage is the total number of studies or PROM-results for which response shift was not detected

**Design-based methods:** Most studies (N = 98) applied design-based methods to investigate response shift, of which the then-test was the most common method (N = 84 or 86%; see Table 3). The total number of then-test studies that investigated impact was 53 (63%; with N = 9 alternative approaches, see Table 4), where information about impact was available (or could be calculated) for 50 of these studies (cf. [40]). Only 10 then-test studies (12%) did not investigate impact and did not provide information on pre-post and then-post comparisons (cf. [41]). For a relatively large number of then-test studies (i.e., N = 27 or 32%; including 21 studies that did not investigate impact (see Table 3), and 6 studies that investigated impact using alternative approaches), information on (un)adjusted change scores was available (or could be calculated) but were not explicitly compared or described [cf. [42]). Thus, information about impact was available for 71 (85%) then-test studies in total (see Table 6). Evidence of impact of response shift was indicated in 79% of these studies, where 59% and 61% of studies evidenced impact on statistical significance and/or magnitude of change in PROM-scores, respectively. Evidence of impact on decisions was indicated in only 8% of studies.

Of the 24 response shift studies that applied another design-based method (individualized or other methods), there were five studies that used alternative approaches to investigate impact (see Table 4) but did not provide information about impact (see Table 3). There were only two studies (17%) that investigated impact and provided information about impact [43, 44]; one of those studies [43] also evidenced impact of response shift. Most studies that used individualized or other design-based methods (i.e., N = 17 or 71%) did not investigate impact and did not provide information about impact (see Table 3).

**Latent variable methods:** Of the 58 response shift studies that applied latent variable methods (LVMs), the majority applied SEM (N = 56 or 97%; see Table 3). Information about impact could not be derived from the statistics provided for any of the SEM-studies that did not explicitly investigate impact of response shift (N = 25; 45%) or that investigated impact in other ways (N = 12; 21%). There were 2 studies (4%) that investigated impact in a way that was consistent with our operationalization, but that did not provide statistical information to derive evidence of impact. Therefore, information about impact was available for 17 SEM-studies (30%), which is a relatively lower number compared to the overall study-level (51%) and then-test (85%) results. Evidence of impact was also indicated in relatively lower number of studies (i.e., 29% for SEM-studies compared to 69% for the overall study-level and 79% for then-test studies; see Table 6).

Of the four LVM-studies that applied IRT or Rasch methods, there were three studies that investigated impact, of which two studies [45, 46] also provided information on impact of response shift (see Table 3) but did not find evidence of impact of response shift (see Table 6). One study used an alternative approach to investigate impact (see Table 4).

**Other response shift methods:** None of the response shift studies that applied regression methods (N = 27), or study-specific methods (N = 5), provided information about impact of response shift (see Table 6). Some studies that applied regression-based methods investigated impact using alternative approaches (see Table 4), but evidence of impact of response shift could not be evaluated for these studies.

### Impact of response shift: PROM-results

A total of 47 studies (27%) investigated response shift in only one target construct with one PROM in a single sample and with one response shift method; resulting in a single effect per study that can be impacted by response shift. Most studies, however, reported on multiple effects (or PROM-results). Specifically, there were 69 studies (40%) that reported between 2–5 effects, 32 studies (18%) reported between 6–10 effects, and 25 studies (14%) reported between 11–44 effects. A total of 943 PROM-results were available for the evaluation of impact of response shift.

The results at the level of the PROM-results show that the percentages of effects for which impact was investigated, information about impact was available, and impact was evidenced are generally lower than at the study-level, overall and across response shift methods (see Table 9). Notably, impact of response shift was evidenced in 69% of studies and only 41% of the PROM-results. The overall pattern of differences across response shift methods appears similar compared to study-level results. Results on response shift *detection* versus *impact* of response shift (see Table 9) showed that of the 265 PROM-results for which response shift was detected 118 effects (45%) did not show evidence of impact of response shift, whereas out of 237 PROM-results where no significant response shift was detected, 60 effects (25%) did show evidence of impact of response shift.

**Table 9:** Number of PROM-results for which impact was investigated, information about impact was available, and impact was evidenced; overall and per response shift method

	Total #nr of effects	Impact investigated^3^	Information available	Impact Evidenced			
				Overall^4^ N (%)^2^	Significance N (%)^2^	Magnitude N (%)^2^	Decision N (%)^2^
	N	N (%)^1^	N (%)^1^				
Design-based methods:	592						
Then–test	519	296 (57%)	417 (80%)	198 (47%)	96 (23%)	154 (37%)	10 (2%)
Individualized methods	27	16 (59%)	8 (30%)	2 (25%)	1 (13%)	1 (13%)	0 (0%)
Other methods	46	3 (7%)	0 (0%)	–	–	–	–
Latent variable methods:	238						
SEM	233	115 (49%)	75 (32%)	7 (9%)	2 (3%)	3 (4%)	2 (3%)
IRT/RMT	5	3 (60%)	2 (40%)	0 (0%)	0 (0%)	0 (0%)	0 (0%)
Regression methods:	108						
With classification	31	2 (7%)	0 (0%)	–	–	–	
Without classification	77	11 (14%)	0 (0%)	–	–	–	–
Other study–specific methods	5	0 (0%)	0 (0%)	–	–	–	–
Overall	943	446 (47%)	502 (53%)	207 (41%)	99 (20%)^5^	158 (31%)^6^	12 (2%)

SEM; structural equation modeling, IRT/RMT; Item response theory/Rasch measurement theory. ^1^The denominator for the percentage is the total number of studies/effects in the same row. ^2^The denominator for the percentage is the number of studies/effects that provided information about impact from the same row. ^3^Note that for some effects impact was investigated but information about impact was not provided, and vice versa (see Table 3 for study–level decomposition). ^4^Impact evidenced in terms of a difference in statistical significance, magnitude and/or decisions before and after taking response shift into account. ^5^N = 75 results became statistically significant after taking response shift into account. ^6^N = 115 results became of larger size after taking response shift into account

Of the 502 PROM-results for which information about impact was available (see Table 8), there were 99 (20%) PROM-results with impact on statistical significance, with 75 PROM-results (76% out of N = 99) becoming statistically significant after taking response shift into account. Of the 158 PROM-results (31%) with impact on magnitude, there were 115 effects (73% out of N = 158) that became of larger size after taking response shift into account. There were only 12 effects (2%) for which there was impact on decisions made based on the PROM-result (see Table 7 for illustrative examples). For two effects, from two studies, the direction of change was different after taking response shift into account [7;47]. For another two effects from one study [21] the treatment was only cost-effective when taking response shift into account, and for the remaining eight effects from four studies [48–51] there was impact on the interpretation of treatment-effectiveness. Specifically, taking response shift into account changed the conclusion about symptoms remaining elevated [48] and comparisons between treatment groups were only statistically significant after taking response shift into account [49–51].

## Discussion

This systematic review builds on our previous work regarding the prevalence and magnitude of response shift [5; 11] that included all longitudinal quantitative response shift studies using PROMs, until May 2023. The current study addresses the important additional question of whether response shift impacts the conclusions about change in PROs.

### Investigation of impact of response shift

About half (55%) of the evaluated studies investigated impact of response shift, and two-thirds of those studies did so by comparing change in PROMs before and after taking response shift into account. Alternative impact investigations included regression-based analyses to relate response shift to changes in PROM-results or other variables (cf. [24]) and subgroup analyses (cf. [35]) to investigate which individuals were most prone to response shift. For LVM-methods there were a relatively large number of studies that investigated impact at the level of the observed indicators (e.g., individual item- or subscale-scores) rather than at the level of the target construct(s) or PRO(s) (cf. [32]). These studies provide meaningful information (e.g., for which subscale or outcome or for which people response shift is most relevant), and the fact that impact is addressed in 55% of studies is promising. Still, there were many studies that did not address impact of response shift. Moreover, the heterogeneity in assessing impact of response shift indicates that a common framework for impact evaluation is lacking. Our review contributes to such a framework by explicating a systematic approach with operationalizations for evaluating impact. Since PROM-results are used to inform healthcare decision-making, a framework for the evaluation of impact of response shift on such decisions is an important way forward for the field of response shift research [52].

### Information about impact of response shift

Information about impact of response shift on PROM-results was available for about half of the number of studies (i.e., 51%). Although the number of times impact was investigated was similar across response shift methods, the availability of information on impact was relatively high for the then-test method (85% of then-test studies) as compared to other response shift methods (0–50%). Therefore, the evaluation of evidence of impact was largely based on results from then-test studies, i.e., 71 out of 89 studies (80%) and 417 out of 502 effects (83%). As the validity of the then-test method has been questioned (cf. [53; 54; 55]), the results regarding impact of response shift should be interpreted with caution.

### Evidence of impact of response shift

About two-thirds (69%) of response shift studies for which information about impact was available also evidenced impact of response shift. The evidence of impact at the level of PROM-results – less than half of the effects (i.e., 41%) – was more modest. The type of inferences that changed when taking response shift into account were mostly about the statistical significance and/or magnitude. Although such changes may impact *conclusions* about change in PROM-scores, evidence of impact on *decisions* made based on these same results was provided for only 8% of studies and 2% of effects. That is, even though changes in PROM-scores may become statistically significant or of larger magnitude after adjusting for response shift, the consequences for the decisions made based on these results were infrequently considered. However, lack of evidence of impact on decisions may (partly) be due to lack of reporting on impact on decisions in response shift studies.

Our results with regards to evidence of impact of response shift at the study-level (i.e., 69% of N = 89 studies) are comparable to results from Schwartz et al. [9] (i.e., 70% of N = 10 studies). A strength of our study is that we included studies with all known quantitative response shift methods and a wide variety of population-, study-, and PROM-characteristics. Another strength is that we used an explicit operationalization of impact, where we evaluated differences in statistical significance and magnitude of change, which can be objectively assessed. This enables an evaluation of impact beyond observing greater or smaller change after taking response shift into account. Whereas rules of thumb to determine whether effects are meaningful according to statistical conventions are helpful (i.e., statistically significant or of small/medium/large size), they also provide a limited view on what is “meaningful”. Therefore, the evaluation of impact on decisions made based on PROM-results was an important additional focus of the current study. Even though impact on decisions was rarely addressed (in only 8% of studies), some laudable examples include decisions about (cost-) effectiveness of treatment (cf. [21] ) or comparison of treatment groups (cf. [50]). Moreover, impact on decisions can be determined even for methods that do not allow for evaluations of magnitude or statistical significance, therefore allowing for a more comprehensive evaluation of impact of response shift.

Comparison between *detection* of response shift and *impact* of response shift showed that detection of response shift did not necessarily result in evidence of impact of response shift (i.e., in 27% of studies and 45% of PROM-results there was no impact although there was (statistically significant) response shift detected), whereas a lack of response shift detection could still coincide with impact (i.e., 43% of studies and 25% of PROM-results for which there was no response shift detected did show evidence of impact of response shift). These results empirically substantiate that evaluation of impact of response shift provides important additional information to detection of response shift alone. Furthermore, when impact on statistical significance or magnitude was evidenced, PROM-results most often became significant or of larger size after taking response shift into account. However, as interpretation of direction of effects might not be consistent across studies (e.g., larger change in PROM-scores may not always be desired) these results should be interpreted with caution.

### Other limitations

The results on impact of response shift are descriptive and therefore do not warrant explicit (causal) inferences regarding the comparisons made. The availability of response shift studies might be influenced by publication bias, reporting biases (e.g., when statistical information to derive impact is mostly missing in studies that lack evidence of impact) or reporting conventions (e.g., availability of information may depend on reporting conventions for specific methods). Another limitation is that it was not possible to consistently differentiate impact of response shift arising from the different types of response shift effects (i.e., recalibration, reprioritization and/or reconceptualization). Finally, our review did not include qualitative response shift research. Qualitative approaches could offer a valuable inquiry into response shift, and possibly impact of response shift, but were outside the scope of the current study.

### Conclusion and future directions

The current review provides a systematic approach with operationalizations for evaluating impact, that can be applied to all response shift studies. The comprehensive overview of impact of response shift shows that impact is investigated in about half of the response shift studies. Statistical significance and/or magnitude of PROM-results are most often impacted, whereas impact on decisions is rarely addressed. We recommend future reports of response shift to include *impact* as an addition to the previously published recommendations on the standardized reporting of response shift results (e.g. [9; 11 ;52; 56]). A unified framework for the evaluation of impact of response shift in the context of healthcare decision-making is an important way forward for the field. This is crucial for valid interpretation of PROM-results, and thus valid conclusions and decisions made about change in PROs.

## Data Availability

The data analyzed for the current study are available at https://osf.io/q9erk/.
