# Correction: Impact of response shift on change in patient-reported outcomes: a systematic review

**DOI:** 10.1007/s11136-026-04329-2

**Published:** 2026-08-26

**Authors:** Mathilde G. E. Verdam, Jae-Yung Kwon, Lara Russell, Véronique Sébille, Mirjam A. G. Sprangers, Richard Sawatzky, The Response Shift –in Sync Working Group

**Affiliations:** 1 Department of Methodology and Statistics, Institute of Psychology, Leiden University, Leiden, The Netherlands; 2 Medical Psychology, Amsterdam UMC Location University of Amsterdam, Amsterdam, The Netherlands; 3 School of Nursing, Trinity Western University, 7600 Glover Road, Langley, BC V2Y 1Y1 Canada; 4 School of Nursing, University of Victoria, Victoria, BC Canada; 5 Institute On Aging and Lifelong Health, University of Victoria, University of Victoria, Victoria, BC Canada; 6 MethodS in Patient-Centered Outcomes and HEalth ResEarch, CHU Nantes, INSERM, Nantes Université, Université de Tours, SPHERE, Nantes, 44000 France; 7 Amsterdam Public Health, Mental Health, Amsterdam, The Netherlands; 8 Centre for Advancing Health Outcomes, Providence Health Care Research Institute, Vancouver, Canada; 9 Institute of Health and Care Sciences, Centre for Person-Centred Care (GPCC), Sahlgrenska Academy, University of Gothenburg, Gothenburg, Sweden

**Correction to: Quality of Life Research (2026) 35:203** https://doi.org/10.1007/s11136-026-04307-8

In this article, 'The Response Shift –in Sync Working Group' was missing from the author list. And few formatting errors like spacing and indentation were missing in Tables 1, 3, 4, 6, 7 and 9.

Also, the data availability statement was incorrectly given as ‘No datasets were generated or analysed during the current study’ but should have been ‘The data analyzed for the current study are available at https://osf.io/q9erk/’.

The original article has been corrected.

